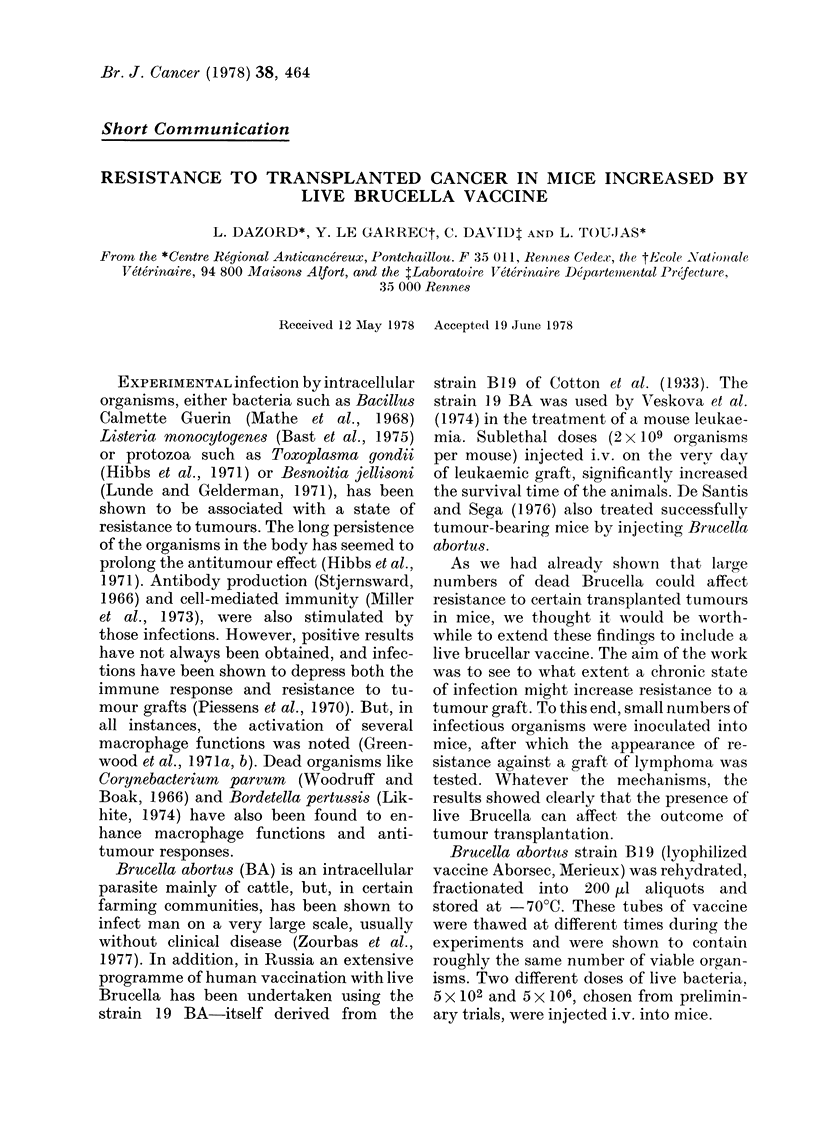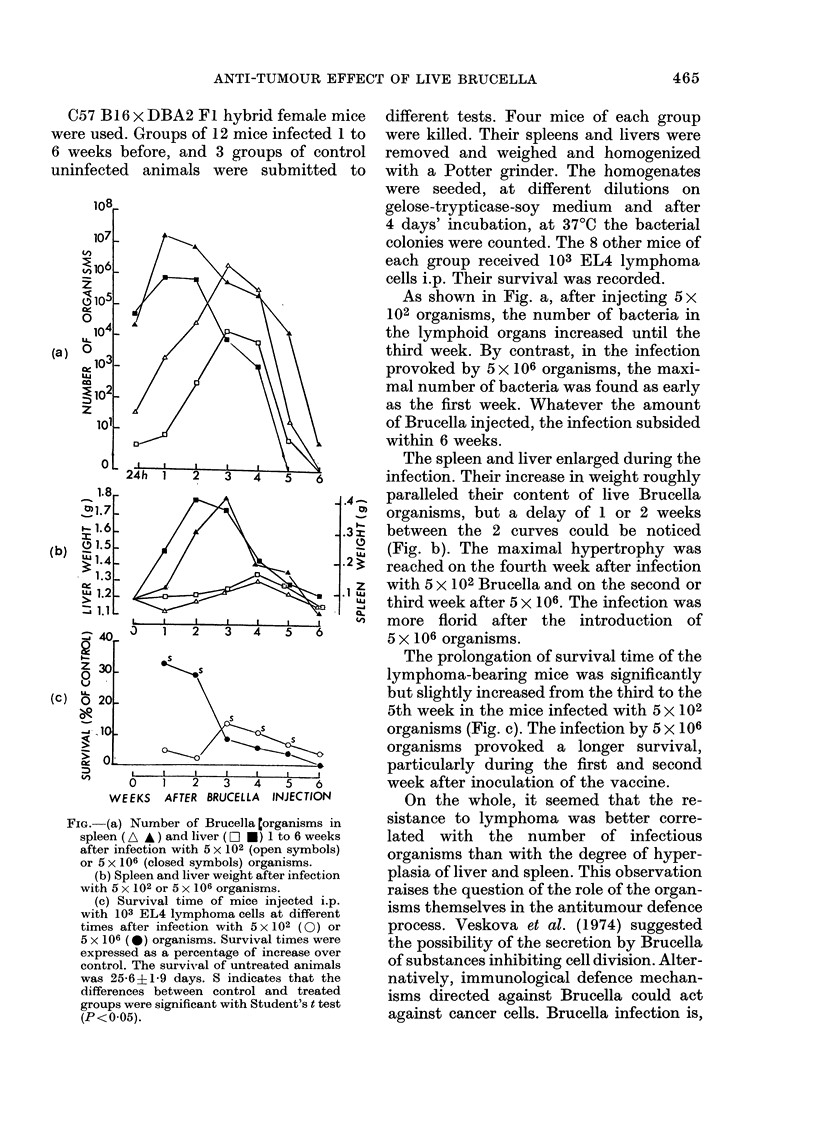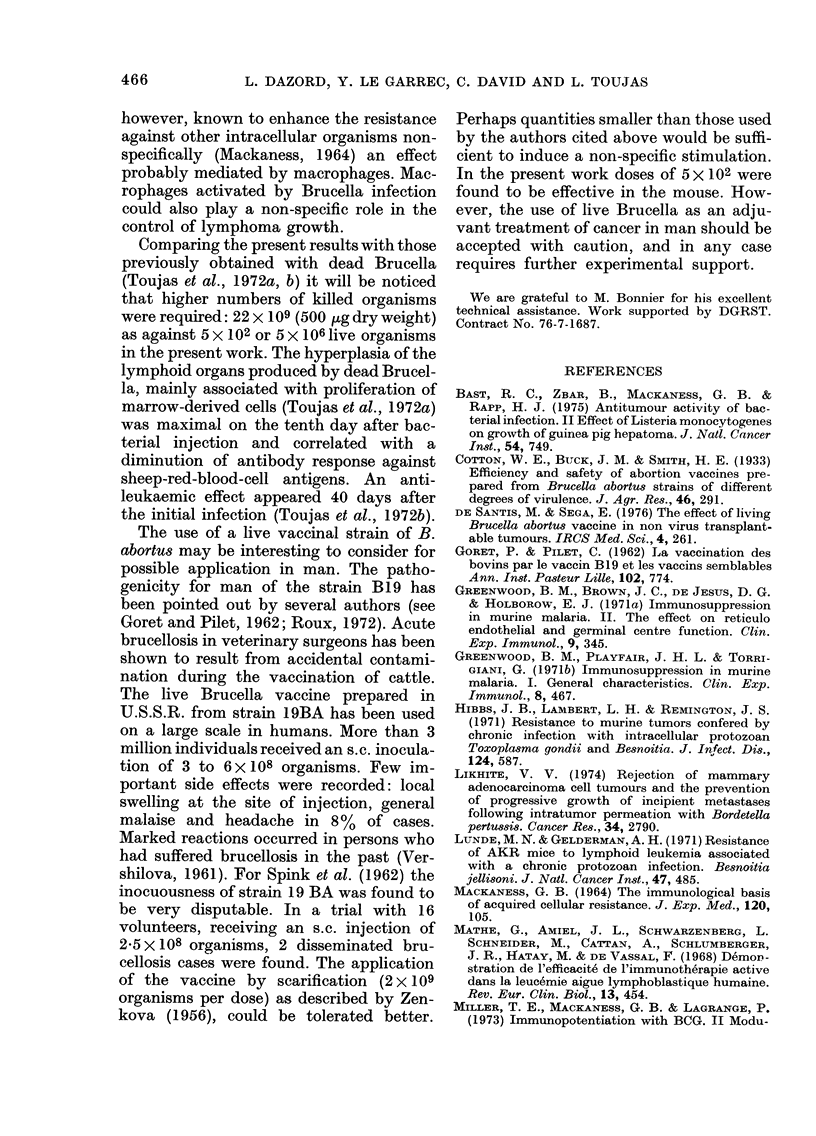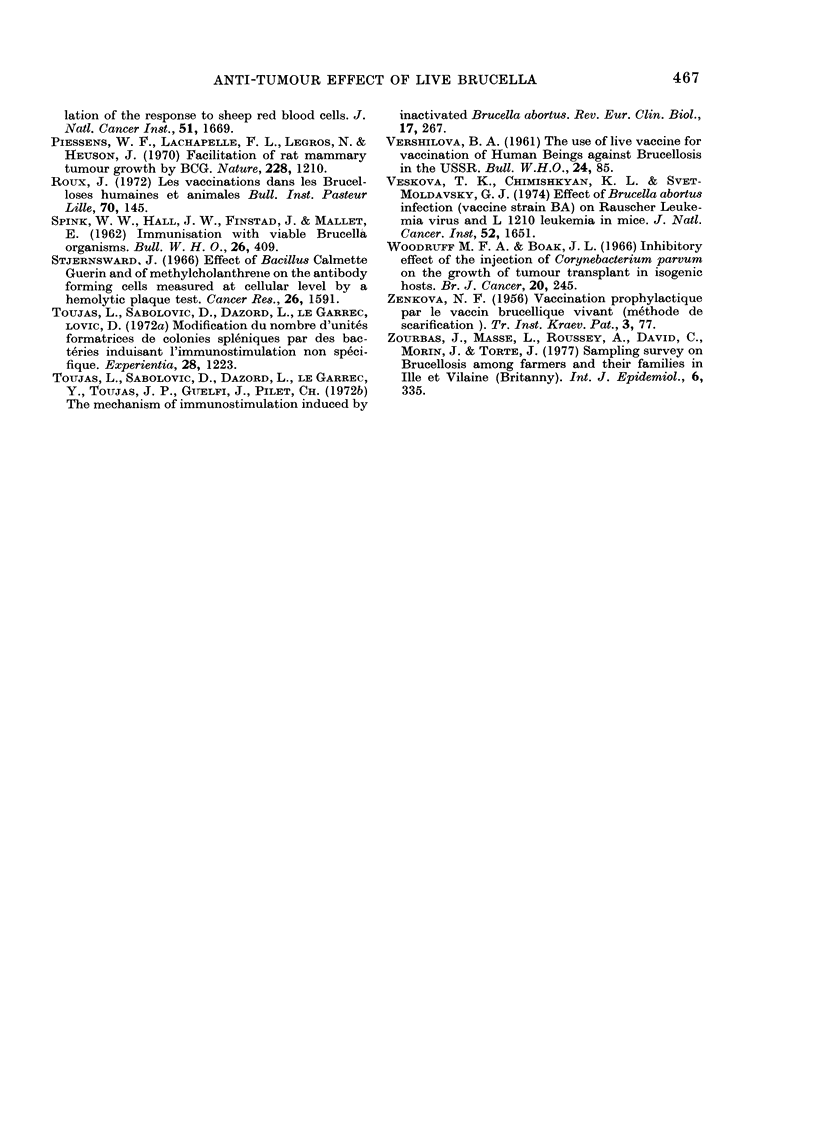# Resistance to transplanted cancer in mice increased by live Brucella vaccine.

**DOI:** 10.1038/bjc.1978.231

**Published:** 1978-09

**Authors:** L. Dazord, Y. Le Garrec, C. David, L. Toujas


Br. J. Cancer (1978) **38**, 464

***Short Communication***

### RESISTANCE TO TRANSPLANTED CANCER IN MICE INCREASED BY LIVE BRUCELLA VACCINE

L. DAZORD*, Y. LE GARREC†, C. DAVID‡ AND L. TOUJAS*

*From the *Centre Régional Anticancéreux, Pontchaillou. F 35 011, Rennes Cedex, the †Ecole Nationale Vétérinaire, 94 800 Maisons Alfort, and the ‡Laboratoire Vétérinaire Départemental Préfecture, 35 000 Rennes*

Received 12 May 1978 Accepted 19 June 1978

EXPERIMENTAL infection by intracellular organisms, either bacteria such as *Bacillus* Calmette Guerin (Mathe *et al.*, 1968) *Listeria monocytogenes* (Bast *et al.*, 1975) or protozoa such as *Toxoplasma gondii* (Hibbs *et al.*, 1971) or *Besnoitia jellisoni* (Lunde and Gelderman, 1971), has been shown to be associated with a state of resistance to tumours. The long persistence of the organisms in the body has seemed to prolong the antitumour effect (Hibbs *et al.*, 1971). Antibody production (Stjernsward, 1966) and cell-mediated immunity (Miller *et al.*, 1973), were also stimulated by those infections. However, positive results have not always been obtained, and infections have been shown to depress both the immune response and resistance to tumour grafts (Piessens *et al.*, 1970). But, in all instances, the activation of several macrophage functions was noted (Greenwood *et al.*, 1971*a*, *b*). Dead organisms like *Corynebacterium parvum* (Woodruff and Boak, 1966) and *Bordetella pertussis* (Likhite, 1974) have also been found to enhance macrophage functions and antitumour responses.

*Brucella abortus* (BA) is an intracellular parasite mainly of cattle, but, in certain farming communities, has been shown to infect man on a very large scale, usually without clinical disease (Zourbas *et al.*, 1977). In addition, in Russia an extensive programme of human vaccination with live Brucella has been undertaken using the strain 19 BA—itself derived from the strain B19 of Cotton *et al.* (1933). The strain 19 BA was used by Veskova *et al.* (1974) in the treatment of a mouse leukaemia. Sublethal doses ($2\times10^9$ organisms per mouse) injected i.v. on the very day of leukaemic graft, significantly increased the survival time of the animals. De Santis and Sega (1976) also treated successfully tumour-bearing mice by injecting *Brucella abortus*.

As we had already shown that large numbers of dead Brucella could affect resistance to certain transplanted tumours in mice, we thought it would be worthwhile to extend these findings to include a live brucellar vaccine. The aim of the work was to see to what extent a chronic state of infection might increase resistance to a tumour graft. To this end, small numbers of infectious organisms were inoculated into mice, after which the appearance of resistance against a graft of lymphoma was tested. Whatever the mechanisms, the results showed clearly that the presence of live Brucella can affect the outcome of tumour transplantation.

*Brucella abortus* strain B19 (lyophilized vaccine Aborsec, Merieux) was rehydrated, fractionated into 200 $\mu$l aliquots and stored at $-70$°C. These tubes of vaccine were thawed at different times during the experiments and were shown to contain roughly the same number of viable organisms. Two different doses of live bacteria, $5\times10^2$ and $5\times10^6$, chosen from preliminary trials, were injected i.v. into mice.

C57 B16 × DBA2 F1 hybrid female mice were used. Groups of 12 mice infected 1 to 6 weeks before, and 3 groups of control uninfected animals were submitted to different tests. Four mice of each group were killed. Their spleens and livers were removed and weighed and homogenized with a Potter grinder. The homogenates were seeded, at different dilutions on gelose-trypticase-soy medium and after 4 days' incubation, at 37°C the bacterial colonies were counted. The 8 other mice of each group received $10^3$ EL4 lymphoma cells i.p. Their survival was recorded.

FIG.—(a) Number of Brucella organisms in spleen (△ ▲) and liver (□ ■) 1 to 6 weeks after infection with $5 \times 10^2$ (open symbols) or $5 \times 10^6$ (closed symbols) organisms.

(b) Spleen and liver weight after infection with $5 \times 10^2$ or $5 \times 10^6$ organisms.

(c) Survival time of mice injected i.p. with $10^3$ EL4 lymphoma cells at different times after infection with $5 \times 10^2$ (○) or $5 \times 10^6$ (●) organisms. Survival times were expressed as a percentage of increase over control. The survival of untreated animals was $25{\cdot}6 \pm 1{\cdot}9$ days. S indicates that the differences between control and treated groups were significant with Student's $t$ test ($P < 0{\cdot}05$).

As shown in Fig. a, after injecting $5 \times 10^2$ organisms, the number of bacteria in the lymphoid organs increased until the third week. By contrast, in the infection provoked by $5 \times 10^6$ organisms, the maximal number of bacteria was found as early as the first week. Whatever the amount of Brucella injected, the infection subsided within 6 weeks.

The spleen and liver enlarged during the infection. Their increase in weight roughly paralleled their content of live Brucella organisms, but a delay of 1 or 2 weeks between the 2 curves could be noticed (Fig. b). The maximal hypertrophy was reached on the fourth week after infection with $5 \times 10^2$ Brucella and on the second or third week after $5 \times 10^6$. The infection was more florid after the introduction of $5 \times 10^6$ organisms.

The prolongation of survival time of the lymphoma-bearing mice was significantly but slightly increased from the third to the 5th week in the mice infected with $5 \times 10^2$ organisms (Fig. c). The infection by $5 \times 10^6$ organisms provoked a longer survival, particularly during the first and second week after inoculation of the vaccine.

On the whole, it seemed that the resistance to lymphoma was better correlated with the number of infectious organisms than with the degree of hyperplasia of liver and spleen. This observation raises the question of the role of the organisms themselves in the antitumour defence process. Veskova *et al.* (1974) suggested the possibility of the secretion by Brucella of substances inhibiting cell division. Alternatively, immunological defence mechanisms directed against Brucella could act against cancer cells. Brucella infection is, however, known to enhance the resistance against other intracellular organisms non-specifically (Mackaness, 1964) an effect probably mediated by macrophages. Macrophages activated by Brucella infection could also play a non-specific role in the control of lymphoma growth.

Comparing the present results with those previously obtained with dead Brucella (Toujas *et al.*, 1972*a*, *b*) it will be noticed that higher numbers of killed organisms were required: $22 \times 10^9$ (500 $\mu$g dry weight) as against $5 \times 10^2$ or $5 \times 10^6$ live organisms in the present work. The hyperplasia of the lymphoid organs produced by dead Brucella, mainly associated with proliferation of marrow-derived cells (Toujas *et al.*, 1972*a*) was maximal on the tenth day after bacterial injection and correlated with a diminution of antibody response against sheep-red-blood-cell antigens. An anti-leukaemic effect appeared 40 days after the initial infection (Toujas *et al.*, 1972*b*).

The use of a live vaccinal strain of *B. abortus* may be interesting to consider for possible application in man. The pathogenicity for man of the strain B19 has been pointed out by several authors (see Goret and Pilet, 1962; Roux, 1972). Acute brucellosis in veterinary surgeons has been shown to result from accidental contamination during the vaccination of cattle. The live Brucella vaccine prepared in U.S.S.R. from strain 19BA has been used on a large scale in humans. More than 3 million individuals received an s.c. inoculation of 3 to $6 \times 10^8$ organisms. Few important side effects were recorded: local swelling at the site of injection, general malaise and headache in 8% of cases. Marked reactions occurred in persons who had suffered brucellosis in the past (Vershilova, 1961). For Spink *et al.* (1962) the inocuousness of strain 19 BA was found to be very disputable. In a trial with 16 volunteers, receiving an s.c. injection of $2{\cdot}5 \times 10^8$ organisms, 2 disseminated brucellosis cases were found. The application of the vaccine by scarification ($2 \times 10^9$ organisms per dose) as described by Zenkova (1956), could be tolerated better. Perhaps quantities smaller than those used by the authors cited above would be sufficient to induce a non-specific stimulation. In the present work doses of $5 \times 10^2$ were found to be effective in the mouse. However, the use of live Brucella as an adjuvant treatment of cancer in man should be accepted with caution, and in any case requires further experimental support.

We are grateful to M. Bonnier for his excellent technical assistance. Work supported by DGRST. Contract No. 76-7-1687.

### REFERENCES

Bast, R. C., Zbar, B., Mackaness, G. B. & Rapp, H. J. (1975) Antitumour activity of bacterial infection. II Effect of Listeria monocytogenes on growth of guinea pig hepatoma. *J. Natl. Cancer Inst.*, **54**, 749.

Cotton, W. E., Buck, J. M. & Smith, H. E. (1933) Efficiency and safety of abortion vaccines prepared from *Brucella abortus* strains of different degrees of virulence. *J. Agr. Res.*, **46**, 291.

de Santis, M. & Sega, E. (1976) The effect of living *Brucella abortus* vaccine in non virus transplantable tumours. *IRCS Med. Sci.*, **4**, 261.

Goret, P. & Pilet, C. (1962) La vaccination des bovins par le vaccin B19 et les vaccins semblables *Ann. Inst. Pasteur Lille*, **102**, 774.

Greenwood, B. M., Brown, J. C., de Jesus, D. G. & Holborow, E. J. (1971*a*) Immunosuppression in murine malaria. II. The effect on reticulo endothelial and germinal centre function. *Clin. Exp. Immunol.*, **9**, 345.

Greenwood, B. M., Playfair, J. H. L. & Torrigiani, G. (1971*b*) Immunosuppression in murine malaria. I. General characteristics. *Clin. Exp. Immunol.*, **8**, 467.

Hibbs, J. B., Lambert, L. H. & Remington, J. S. (1971) Resistance to murine tumors confered by chronic infection with intracellular protozoan *Toxoplasma gondii* and *Besnoitia*. *J. Infect. Dis.*, **124**, 587.

Likhite, V. V. (1974) Rejection of mammary adenocarcinoma cell tumours and the prevention of progressive growth of incipient metastases following intratumor permeation with *Bordetella pertussis*. *Cancer Res.*, **34**, 2790.

Lunde, M. N. & Gelderman, A. H. (1971) Resistance of AKR mice to lymphoid leukemia associated with a chronic protozoan infection. *Besnoitia jellisoni*. *J. Natl. Cancer Inst.*, **47**, 485.

Mackaness, G. B. (1964) The immunological basis of acquired cellular resistance. *J. Exp. Med.*, **120**, 105.

Mathe, G., Amiel, J. L., Schwarzenberg, L. Schneider, M., Cattan, A., Schlumberger, J. R., Hatay, M. & de Vassal, F. (1968) Démonstration de l'efficacité de l'immunothérapie active dans la leucémie aigue lymphoblastique humaine. *Rev. Eur. Clin. Biol.*, **13**, 454.

Miller, T. E., Mackaness, G. B. & Lagrange, P. (1973) Immunopotentiation with BCG. II Modulation of the response to sheep red blood cells. *J. Natl. Cancer Inst.*, **51**, 1669.

PIESSENS, W. F., LACHAPELLE, F. L., LEGROS, N. & HEUSON, J. (1970) Facilitation of rat mammary tumour growth by BCG. *Nature*, **228**, 1210.

ROUX, J. (1972) Les vaccinations dans les Brucelloses humaines et animales *Bull. Inst. Pasteur Lille*, **70**, 145.

SPINK, W. W., HALL, J. W., FINSTAD, J. & MALLET, E. (1962) Immunisation with viable Brucella organisms. *Bull. W. H. O.*, **26**, 409.

STJERNSWARD, J. (1966) Effect of *Bacillus* Calmette Guerin and of methylcholanthrene on the antibody forming cells measured at cellular level by a hemolytic plaque test. *Cancer Res.*, **26**, 1591.

TOUJAS, L., SABOLOVIC, D., DAZORD, L., LE GARREC, LOVIC, D. (1972*a*) Modification du nombre d'unités formatrices de colonies spléniques par des bactéries induisant l'immunostimulation non spécifique. *Experientia*, **28**, 1223.

TOUJAS, L., SABOLOVIC, D., DAZORD, L., LE GARREC, Y., TOUJAS, J. P., GUELFI, J., PILET, CH. (1972*b*) The mechanism of immunostimulation induced by inactivated *Brucella abortus*. *Rev. Eur. Clin. Biol.*, **17**, 267.

VERSHILOVA, B. A. (1961) The use of live vaccine for vaccination of Human Beings against Brucellosis in the USSR. *Bull. W.H.O.*, **24**, 85.

VESKOVA, T. K., CHIMISHKYAN, K. L. & SVET-MOLDAVSKY, G. J. (1974) Effect of *Brucella abortus* infection (vaccine strain BA) on Rauscher Leukemia virus and L 1210 leukemia in mice. *J. Natl. Cancer. Inst*, **52**, 1651.

WOODRUFF M. F. A. & BOAK, J. L. (1966) Inhibitory effect of the injection of *Corynebacterium parvum* on the growth of tumour transplant in isogenic hosts. *Br. J. Cancer*, **20**, 245.

ZENKOVA, N. F. (1956) Vaccination prophylactique par le vaccin brucellique vivant (méthode de scarification ). *Tr. Inst. Kraev. Pat.*, **3**, 77.

ZOURBAS, J., MASSE, L., ROUSSEY, A., DAVID, C., MORIN, J. & TORTE, J. (1977) Sampling survey on Brucellosis among farmers and their families in Ille et Vilaine (Britanny). *Int. J. Epidemiol.*, **6**, 335.